# Preservation of sputum samples with cetylpyridinium chloride (CPC) for tuberculosis cultures and Xpert MTB/RIF in a low-income country

**DOI:** 10.1186/s12879-017-2642-z

**Published:** 2017-08-04

**Authors:** Hellen Hiza, Basra Doulla, Mohamed Sasamalo, Jerry Hella, Lujeko Kamwela, Francis Mhimbira, Klaus Reither, Sebastien Gagneux, Levan Jugheli, Lukas Fenner

**Affiliations:** 1 Ifakara Health Institute, Bagamoyo, Tanzania; 2 National Tuberculosis and Leprosy Programme, Dar es Salaam, Tanzania; 3 Swiss Tropical and Public Health Institute, Basel, Switzerland; 4 University of Basel, Basel, Switzerland; 5 Institute of Social and Preventive Medicine, University of Bern, Bern, Switzerland

**Keywords:** *Mycobacterium tuberculosis*, Tuberculosis, Cetylpyridinium chloride, CPC, Sample transport, Preservation, Contamination, Culture, Sputum, Recovery, Tuberculosis, Low-income country

## Abstract

**Background:**

Culture contamination with environmental bacteria is a major challenge in tuberculosis (TB) laboratories in hot and humid climate zones. We studied the effect of cetylpyridinium chloride (CPC) preservation on culture results and performance of Xpert MTB/RIF.

**Methods:**

Consecutive sputum samples from microscopy smear-positive TB patients were collected. Two-hundred samples were equally split in two aliquots, one aliquot was treated with CPC and stored at ambient temperature for 7 days. The second aliquot was immediately processed. Samples were decontaminated for 20, 15 or 10 min, and subsequently cultured on Löwenstein-Jensen medium. Furthermore, 50 samples were stored for 7, 14 and 21 days, and 100 CPC-pretreated samples tested by Xpert MTB/RIF.

**Results:**

CPC pretreated samples showed a higher culture yield compared to non-treated sputum samples across all decontamination times: 94% vs. 73% at 10 min (*p* = 0.01), 94% vs. 64% at 15 min (*p* = 0.004), and 90% vs. 52% at 20 min (*p* < 0.001). The quantitative culture grading was consistently higher in CPC treated compared to non-CPC treated samples. The proportion of contaminated cultures was lower in CPC pretreated samples across all decontamination times (range 2-6%) compared to non-CPC treated samples (15-16%). For storage times of CPC treated samples of 7, 14, and 21 days, 84, 86, and 84% of the respective cultures were positive. Of 91 CPC treated samples with a positive culture, 90 were also Xpert MTB/RIF positive.

**Conclusions:**

CPC increases culture yield, decreases the proportion of contamination, and does not alter the performance of Xpert MTB/RIF.

## Background

Tanzania is among the 30 countries with the highest tuberculosis (TB) burden worldwide according to the World Health Organization (WHO), with 63,151 TB cases in 2015 [1]. Of these, 60,563 were new cases, and 1580 were previously treated [1]. Retreatment cases are of particular concern to public health due to the failure of treatment with first-line drugs that makes it crucial to closely monitor TB cases, which includes drug susceptibility testing (DST) performed by a TB laboratory. In Tanzania, the central tuberculosis referral laboratory (CTRL) is responsible to perform DST nationwide. DST relies in turn on sputum samples, but these often do not reach the CTRL or, when they do, samples show overgrowth with other bacteria such as nontuberculous mycobacteria from the environment or commensal bacteria from the airways [2, 3]. To correctly identify a positive sputum sample, viability must be preserved and contamination with environmental and commensal bacteria suppressed during transportation [4, 5]. Cetylpyridinium chloride (CPC) with sodium chloride is a simple reagent with low toxicity to *Mycobacterium tuberculosis* that has a mild decontamination effect on bacteria and fungi [6]. The use of Difco neutralization buffer with CPC treated samples before inoculation has been observed to further increase the culture yield [7].

Though CPC can preserve sample viability and reduce contamination, which makes it an effective reagent for storage of sputum samples at ambient temperature for several days [6, 8], rigorous evaluation of the effect of CPC treatment on culture and molecular test performance has been limited in sub-Saharan Africa. We aimed to systematically study the effect of CPC treatment and decontamination time on culture results compared to non-CPC samples, tested if the use of Difco neutralization buffer with CPC treated samples before inoculation can further increase the culture yield, and investigated the performance of Xpert MTB/RIF in CPC treated samples.

## Methods

### Study setting and study procedures

We included 200 sputum samples prospectively collected from an ongoing cohort study of adult smear-positive TB patients (≥18 years) in Dar es Salaam, Tanzania (TB-DAR). Consecutive sputum samples with a volume of >4 mL were transported daily from the TB clinic in Dar es Salaam to the TB laboratory in Bagamoyo (Ifakara Health Institute, Tanzania) using temperature-controlled cooling boxes (4-8 °C), because proper procedures of sputum homogenization and splitting into two aliquots were not possible on site. Sputum microscopy was performed on site using fluorescent light-emitting diode (LED) microscopy, and the quantitative scoring system was based on the number of acid-fast bacilli (AFB) according to the national guideline [9]. Sputum smear-positivity was defined as at least scanty on the quantitative grading system.

### Sputum sample processing

**Fig. 1:**
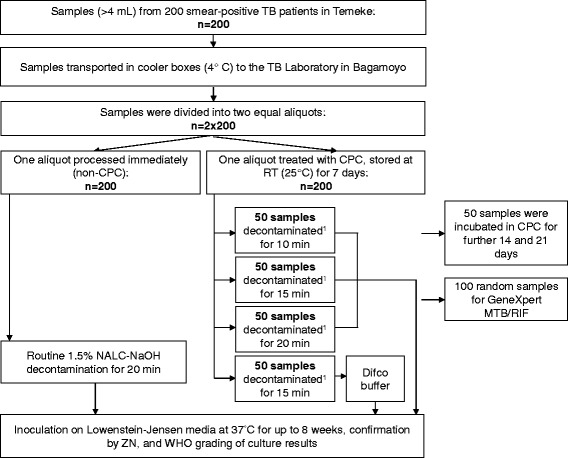
Overview of sputum sample processing. Cetylpyridinium chloride (CPC); Difco buffer, Difco neutralizing buffer; RT, room temperature; TB, tuberculosis; ZN, Ziehl-Nielsen staining. ^1^ 1% NaOH decontamination

The sample processing at the TB laboratory is summarized in Fig. 1.

#### Sputum homogenization and splitting into two aliquots

Sputum samples (>4 mL volume) from the TB clinic were homogenized using sterile Pasteur pipettes and split into two 2 mL aliquots, each in a 50 ml sterile Falcon tube (Fisher Scientific, Switzerland).

#### CPC processing and decontamination of CPC treated samples (first aliquot)

The first aliquot of the same sample was treated with 1% CPC (1:1 CPC and sample), mixed thoroughly using a vortex mixer until an even solution was observed, then incubated at 25 °C for 7, 14, or 21 days before decontamination. The CPC solution was prepared according to the manufacturer’s instruction (Sigma-Aldrich, Switzerland). Following the CPC incubation, samples were decontaminated using 2% NaOH, with 1% NaOH as the final concentration.

For the samples incubated for 7 days we used three different decontamination durations of 10, 15, and 20 min. The decontamination time of the samples incubated for further 14 and 21 days was 15 min.

#### Decontamination of non-CPC samples (second aliquot)

The second aliquot containing native sputum was immediately processed by adding 3% NaOH with 0.4 g of N-acetyl-L-cysteine (NALC), with a 1.5% final concentration of NaOH. The aliquot was digested for 20 min at room temperature (25 °C). Processing was stopped with pH 6.7 phosphate buffer solution (PBS). The mixture was then centrifuged at 4 °C at 3000 g for 20 min to obtain a concentrated pellet, which was reconstituted with three drops of PBS solution and which was directly inoculated on Lowenstein-Jensen (LJ) slants. Cultures were incubated for up to 8 weeks at 37 °C and observed weekly for growth.

#### Addition of neutralization buffer

In 50 samples treated for CPC during 7 days (optimal decontamination time, Table 1), after decontamination a Difco neutralizing buffer (Beckton Dickson, USA) was added instead of PBS buffer. The Difco buffer (containing monopotassium phosphate, sodium thiosulfate, and aryl sulfonate complex) was used to halt further reaction of NaOH and for the resuspension of the pellet. The buffer was prepared by dissolving 5.2 g of Difco powder in 1 L of sterile water and the solution was autoclaved at 121 °C for 15 min. The final buffer pH was 7.2. Difco buffer solution was added up to the 50 mL mark on the falcon tube, and the mixture was centrifuged at 4 °C at 3000 g for 20 min to obtain a concentrated pellet, which was reconstituted with three drops of Difco solution and which was inoculated on LJ slants. Cultures were incubated for up to 8 weeks at 37 °C and observed weekly for growth.

**Table 1:** Effect of decontamination time on culture results in cetylpyridinium chloride (CPC) treated and non-CPC samples from tuberculosis (TB) patients in Tanzania

Culture grading (WHO)	Decontamination of CPC treated samples, *n* (%)					
	20 min^1^		15 min^2^		10 min^3^	
	Non-CPC	CPC	Non-CPC	CPC	Non-CPC	CPC
Contaminated	7 (14)	1 (2)	8 (16)	1 (2)	7 (14)	0 (0)
Negative	17 (34)	4 (8)	8 (16)	2 (4)	6 (12)	3 (6)
Positive	26 (52)	45 (90)	34 (64)	47 (94)	37 (74)	47 (94)
< 20 colonies	2 (4)	3 (6)	2 (4)	2 (4)	0 (0)	0 (0)
1+	5 (10)	7 (14)	11(22)	9 (18)	4 (8)	7 (14)
2+	10 (20)	17 (34)	11 (22)	18(36)	12(24)	15 (30)
3+	9(18)	18 (36)	10 (20)	18 (36)	21(42)	25 (50)
Total	50 (100)	50 (100)	50 (100)	50 (100)	50 (100)	50 (100)

Difference between CPC treated samples and non-CPC according to WHO culture grading (contaminated, negative, positive): *p* < 0.001^1^, *p* = 0.004^2^, and *p* = 0.007^3^, respectively (Fisher’s exact test)

Culture results of 150 sputum samples split into two equal aliquots, one of which was treated with CPC before processing, with varying times of decontamination

#### Mycobacterial cultures

All decontaminated samples were inoculated on the LJ media, incubated at 37 °C and observed for 8 weeks for any growth, with culture grading using the WHO system of <20 colonies, 1+, 2+, 3+ for positive, negative, and contamination. All LJ slants were read every week to observe culture growth.

#### Xpert MTB/RIF testing

For the molecular analysis, 100 CPC treated and decontaminated pellets were randomly selected and tested using the Xpert MTB/RIF assay (Cepheid, USA), in which 0.5 mL of CPC stored pellet was added to 1.5 mL of Xpert buffer. Tubes were vortexed and incubated for 10 min at room temperature, vortexed again and incubated further for 5 min. The suspension was then transferred to the Xpert cartridge and loaded into the Xpert MTB/RIF machine for the analysis of the sample. Results were read and printed two hours after initiating the assay.

### Data analysis

We used descriptive statistics to present the results. The Fisher’s exact test was applied to test the statistical significance of the observed differences in CPC treated and non-CPC samples with different decontamination times (20, 15 and 10 min), and different CPC incubation times (non-CPC, CPC incubation of 7, 14, and 21 days). All analyses were performed in Stata version 14 (Texas, USA).

## Results

### Culture performance of CPC treated and non-CPC samples

#### Effect of CPC treatment and decontamination time on culture recovery

We analyzed 200 samples split in two equal aliquots (2 × 200, Fig. 1), and each treated with or without CPC. Comparing CPC treated with non-CPC samples, we observed a culture positivity of 90% vs. 52% for the 20-min decontamination time (*p* < 0.001), 94% vs. 64% for the 15-min decontamination time (*p* = 0.004), and 94% vs. 74% for the 10-min decontamination time (*p* = 0.007), as shown in Table 1. Contamination rates were 2% vs 14% for the 20 -min decontamination time, 2% vs 16% for the 15-min decontamination time, and 0% vs 14% for 10-min decontamination time (Table 1). The 15-min decontamination showed a low negativity of 4% and the contamination rate was 2% (Table 1).

#### Effect of the duration of CPC treatment on culture recovery

The effect of CPC treatment on culture recovery was examined in 50 sputum samples at three intervals lasting 7, 14, and 21 days (Fig. 1). Culture recovery was 84% (42 samples), 86% (43), and 82% (41) respectively (Table 2). The contamination proportion at the same time intervals was 12% (6 samples), 12% (6), and 6% (3), respectively.

**Table 2:** Effect of CPC incubation time on TB culture recovery

Culture grading (WHO)	Non-CPC	CPC incubation time			
*n* (%)		7 days	14 days		21 days
Contaminated	9 (18)	6 (12)	6 (12)	3 (6)	
Negative	8 (16)	2 (4)	1 (2)	6 (12)	
Positive	33 (66)	42 (84)	43 (86)	41 (82)	
< 20 colonies	0 (0)	3 (6)	4 (8)	3 (6)	
1+	3 (6)	7 (14)	7 (14)	7 (14)	
2+	10 (20)	13 (26)	13 (26)	15 (30)	
3+	20 (40)	19 (38)	19 (38)	16 (32)	
Total	50 (100)	50 (100)	50 (100)	50 (100)	

Decontamination time of 15 min for all CPC treated samples before culture step

Difference between non-CPC and CPC incubation time (7, 14 and 21 days) according to WHO culture grading (contaminated, negative, positive): *p* = 0.077 across groups (Fisher’s exact test)

Culture results of 50 sputum samples not pretreated (non-CPC) or pretreated with CPC before processing (CPC), with varying CPC incubation time of 7, 14, and 21 days

#### Effect of neutralization buffer on culture recovery

In 50 samples, each with two aliquots (2 × 50, Fig. 1), following 7 days of CPC treatment, the Difco buffer did not yield higher culture recovery than the phosphate buffer solution. Recovery was 42/50 (84%) with Difco, and 45/50 (90%) with PBS.

### Performance of Xpert MTB/RIF in CPC treated samples

Of 100 CPC treated samples, 97 were Xpert-positive and three were Xpert-negative (Table 3). Of the 91 culture-positive samples, 90 were Xpert-positive and one Xpert-negative (sensitivity: 98.9%, 95% confidence interval [CI] 94-100%). Of the six culture-negative samples, four were Xpert-positive and two Xpert-negative. All contaminated samples were Xpert-positive (Table 3).

**Table 3:** Effect of CPC pretreatment on the performance of Xpert MTB/RIF

Culture grading (WHO)	Xpert-positive	Xpert-negative
*n*		
Contaminated	3	0
Negative	4	2
Positive	90	1
< 20 colonies	5	0
1+	14	0
2+	30	0
3+	41	1
Total	97	3

Xpert tests results of 100 samples treated with CPC

## Discussion

Our study conducted in the high TB incidence country Tanzania using 200 samples, equally split into a CPC and non-CPC study arm, showed that preservation of sputum samples in CPC increases the TB culture yield and decreases contamination, and that CPC pretreatment has no negative effect on the performance of Xpert MTB/RIF.

Sputum samples can lose viability during long-duration transportation over large distances, which increases contamination by environmental and commensal bacteria present in the sputum and suppresses growth of TB [6, 10]. CPC is known to be an effective sputum transportation reagent, which decreases contamination by microflora during transportation. However, this has been reported only in studies outside of sub-Saharan Africa [5, 10–12]. Sub-Saharan African countries like Tanzania have a distinct hot and humid climate with a high chance of environmental bacteria to contaminate solid TB cultures [5]. In contrast to previous studies [4, 5, 7, 10, 11, 13], we prospectively collected and split the sputum samples into two equal aliquots which allowed a direct comparison between CPC treated and non-CPC samples. Our study also extended sputum sample storage with CPC beyond the usually reported 8 days [4, 10, 11, 13] to systematically investigate the CPC storage time of 14 and 21 days. Finally, we also tested the effect of CPC treatment on the performance of the molecular assay Xpert MTB/RIF.

The CPC treated samples showed higher culture recovery and lower contamination compared to non-CPC samples, with no negative effect on sample viability even when stored at ambient temperature (25 °C) for up to 21 days. Although TB culturing is resource-intensive and require expensive laboratory facilities, TB cultures remain essential for drug susceptibility testing, drug resistance surveillance and molecular epidemiological studies [2, 3]. In our study, 15 min of sample decontamination with 1% NaOH yielded optimal culture recovery, contamination, and culture negativity rates. CPC treated samples should be preferably decontaminated using a lower NaOH concentration than the NaOH concentration routinely used for decontamination (1% versus 1.5%) due to the weak decontaminating properties of CPC [4, 11]. We observed that sputum samples stored with CPC as a preservation method can be stored up to 21 days at room temperature in CPC without reducing the culture recovery rate, consistent with the finding from a previous study conducted in a region of the former Soviet Union [4]. However, the latter study did not systematically test the effect of storage time on culture yield, but used a range of 7 to 36 days between sample collection and processing.

Surprisingly, we did not observe a superior culture recovery using the neutralizing Difco buffer compared to the standard PBS buffer procedure. The Difco buffer possibly neutralizes the weak anti-bacterial activity of CPC, which might have a negative effect on the culture yield. Our finding is in contrast to a previous study which found that neutralization of CPC treated sputum samples showed a higher culture yield compared to the standard PBS procedure [7]. This difference might be partially explained by the different sampling method (random assignment to CPC or non-CPC treatment versus splitting of sputum samples in our study), and the different decontamination time used (10 min versus 15 min in our study) [7].

We did not observe a negative effect of CPC treatment on the performance of the molecular test assay Xpert MTB/RIF. Xpert is a semi-automated molecular assay which detects both *M. tuberculosis* complex species and rifampicin drug resistance [14]. It has been endorsed by WHO and is currently being scaled-up in high TB incidence countries [15]. Our results support the current WHO guidelines for surveillance of drug resistance in TB stating that sputum samples can also produce reliable results when tested using Xpert MTB/RIF even after a month of CPC sputum sample storage [16]. Four samples had a positive Xpert MTB/RIF results, but the sputum cultures were either contaminated or negative. However, as all samples were smear microscopy positive they were likely to be Xpert MTB/RIF true positive. A limitation of this analysis is that we performed Xpert MTB/RIF on CPC treated sputum samples only.

## Conclusion

In conclusion, in sub-Saharan African settings in which sample collection sites can be distant from laboratories CPC, can be used to preserve sample viability during storage and transportation at ambient temperature for at least 7 to as many as 21 days, with a high culture recovery at an optimal decontamination time of 15 min. Furthermore, CPC treated samples can also be used for molecular analysis such as Xpert MTB/RIF. CPC can be used at minimal costs (~0.01 USD per sample) as preservative in sputum samples during transportation in hot humid areas for laboratories and public health surveillance, sample storage, transport using postal services, and for drug resistance surveys. Unfortunately, CPC treated sputum samples can only be cultured on solid media, but not in liquid culture systems such as BACTEC MGIT due to incompatibility with CPC [16, 17].

Future research should focus on the use of CPC or other preservation methods [18] for the use in liquid culture systems such as MIGT as a more sensitive culture detection and more standardized drug susceptibility testing technique than solid culture media. Moreover, addition of neutralizing buffer solutions after pretreatment with CPC to increase culture recovery warrants further exploration.

## Abbreviations

AFB: Acid-fast bacilli
CPC: Cetylpyridinium chloride
CTRL: Central tuberculosis referral laboratory
DST: Drug susceptibility testing
LED: Fluorescent light-emitting diode microscopy
LJ: Lowenstein-Jensen
NALC: N-acetyl-L-cysteine
TB: Tuberculosis
TB-DAR: Ongoing cohort study of adult smear-positive tuberculosis patients in Dar es Salaam, Tanzania
WHO: World Health Organization

## References

[1] WHO. World Health Organization (2015). Global tuberculosis report 2015: country profiles for 22 high burden contries.

[2] Kilale AM, Ngowi BJ, Mfinanga GS, Egwaga S, Doulla B, Kumar AM (2013). Are sputum samples of retreatment tuberculosis reaching the reference laboratories? A 9-year audit in Tanzania. Public Health Action.

[3] Hoza AS, Mfinanga SG, Konig B (2015). Anti-TB drug resistance in Tanga, Tanzania: a cross sectional facility-base prevalence among pulmonary TB patients. Asian Pac J Trop Med.

[4] Pardini M, Varaine F, Iona E, Arzumanian E, Checchi F, Oggioni MR (2005). Cetyl-pyridinium chloride is useful for isolation of *Mycobacterium tuberculosis* from sputa subjected to long-term storage. J Clin Microbiol.

[5] Sankar MM, Kumar P, Munawwar A, Singh J, Parashar D, Singh S (2009). Recovery of *Mycobacterium tuberculosis* from sputum treated with cetylpyridinium chloride. J Clin Microbiol.

[6] Phillips BJ, Kaplan W (1976). Effect of cetylpyridinium chloride on pathogenic fungi and *Nocardia asteroides* in effect of cetylpyridinium. J Clin Microbiol.

[7] Ardizzoni E, Mulders W, Sanchez-Padilla E, Varaine F, de Jong BC, Rigouts L (2014). Decontamination methods for samples preserved in cetylpyridinium chloride and cultured on thin-layer agar. Int J Tuberc Lung Dis..

[8] Selvakumar N, Gomathi Sekar M, Ilampuranan KJ, Ponnuraja C, Narayanan PR (2005). Increased detection by restaining of acid-fast bacilli in sputum samples transported in cetylpyridinium chloride solution. Int J Tuberc Lung Dis.

[9] MoHSW. Manual of the National Tubersculosis and Leprosy Programme in Tanzania. 5th ed; 2006.

[10] Aparna S, Krishna Moorthy KV, Gokhale S (2006). From microscopy centre to culture laboratory a viable ride for *mycobacteria*. Int J Tuberc Lung Dis..

[11] Bhat J, Selvakumar N, Rao VG, Gopi PG, Yadav R, Wares DF (2011). Yield of culture of *Mycobacterium tuberculosis* complex in sputum samples transported from tribal areas. Int J Tuberc Lung Dis.

[12] Selvakumar N, Vanajakumar, Narayana ASL, Suryanarayanan D, Umapathy KC, Paramasvian CN, et al. Use of cetylpyridium chloride for storage of sputum specimens and isolation of *M. tuberculosis.* Indian J Tuberc. 1993;40:95–7.

[13] Tessema B, Beer J, Emmrich F, Sack U, Rodloff AC (2011). Rate of recovery of *Mycobacterium tuberculosis* from frozen acid-fast-bacillus smear-positive sputum samples subjected to long-term storage in Northwest Ethiopia. J Clin Microbiol.

[14] Boehme CC, Nabeta P, Hillemann D, Nicol MP, Shenai S, Krapp F (2010). Rapid molecular detection of tuberculosis and rifampin resistance. N Engl J Med.

[15] WHO (2013). Tuberculosis diagnostics Xpert MTB/RIF test. WHO endorsement and recommendations.

[16] WHO (2015). Guideline for surveillance of drug resistance in tuberculosis.

[17] Lumb R, Ardian M, Waramori G, Syahrial H, Tjitra E, Maguire GP (2006). An alternative method for sputum storage and transport for *M. tuberculosis* drug resistance surveys. Int J Tuberc Lung Dis.

[18] Omar SV, Peters RP, Ismail NA, Jonkman K, Dreyer AW, Said HM (2016). Field evaluation of a novel preservation medium to transport sputum specimens for molecular detection of *Mycobacterium tuberculosis* in a rural African setting. Tropical Med Int Health.

